# New Look and New Title for NIAAA’s Flagship Publication

**Published:** 2012

**Authors:** Kenneth Warren

In 2010, NIAAA marked a significant milestone, celebrating 40 years of research on alcohol abuse and alcoholism. Throughout this time, NIAAA’s journal has played an important role, raising awareness about topics in alcohol research and ensuring that important findings from the field were disseminated to the widest possible audience.

The journal began under the title *Alcohol World.* It had a newsy, magazine-type focus and featured topics of particular interest to a lay audience. In the 1980s, the journal’s content was further honed with the advent of a structured peer-review process. The title also was changed to *Alcohol Health & Research World* to better reflect this research-centered focus. In 1999 that title was further streamlined to *Alcohol Research & Health.* Each issue of the journal was designed to present a sophisticated review of an important topic from the field of alcohol research. The chief goal was to inform scientists from all disciplines on advances taking place in the alcohol research field.

Now, with 40 years behind us, NIAAA is entering a new era of supporting, guiding, and raising awareness of alcohol research. With this new era comes a new resolve to better serve scientists working in the alcohol research field. We recognize that as the journal’s content has evolved, it has become less of a resource for the lay audience and more a review geared to scientists. Its greatest value has been its succinct and timely reviews on topics of interest to alcohol researchers or those interested in findings from this unique and varied field.

This current issue carries a new title, *Alcohol Research: Current Reviews,* which speaks directly to the journal’s substance and scope. The basic content and focus will remain the same. We will continue to provide reviews written by leaders in the field, with each issue focusing on one particular topic in alcohol research. In addition, NIAAA will enhance this content by including an introduction in each issue that provides a historical perspective on the topic as well as where this area of research might be headed in the future. Other new sections include a resources department, designed to provide scientists with tools they can tap for additional information.

This issue on genetics provides an excellent opportunity to showcase these changes. The rapid advances in genetics have implications for nearly every area of alcohol research. One of the most exciting aspects has been the increasing availability of comprehensive datasets that researchers can tap to identify genes related to alcohol abuse, understand the influence of the environment, develop effective new drugs that may allow personalized medical approaches to be realized, and much more. This issue’s resources department offers a comprehensive listing of bioinformation databases in use today, as well as the tools available to analyze that data and guidance on how researchers can use this information to advance their own research efforts.

We hope you find these changes to the journal useful and its articles helpful in keeping you abreast of the vast field of alcohol research. For those of you who are researchers, we hope the new resources department will help you tap into a wider network of collaborators and data that will be of use to you. Look for upcoming issues of the journal to address topics such as stress, the burden of disease, and the state of the fast-moving epigenetics field. We are confident that these changes to the journal will help to ensure that *Alcohol Research: Current Reviews* remains a pre-eminent and trusted resource, chronicling the advances in various fields of alcohol research.

